# One Stop; Addiction, Obstetrics & Perinatal Mental Health Pathway in North East Essex

**DOI:** 10.1192/bjo.2024.502

**Published:** 2024-08-01

**Authors:** Vyasa Immadisetty, Opeyemi Oshingbesan, Sheeba Sarafudheen, Shelley Gurney

**Affiliations:** 1 Essex Partnership University NHS Foundation Trust, Colchester, United Kingdom; 2 East Suffolk and North Essex NHS Foundation Trust, Colchester, United Kingdom

## Abstract

**Aims:**

Addiction services in Essex are provided as a collaborative by NHS run Essex STaRS, Open Roads, SHARP and ARC provide psychosocial care. YPDAS supports the young people.

Observed gap: Pregnant women with addiction problems were running from pillar to post to receive care and support needed during this challenging phase of their life.

The one stop clinic provided an all-encompassing care pathway to fill the above need and improving outcomes for mothers and babies.

**Methods:**

Description:

The new pathway was setup in 2019 on a hub & spoke model. The one stop clinic was at centre, comprising Substance Misuse, Midwifery and Obstetrics. The spokes included Perinatal-mental health, Neonatal, Adult, Child Social services, CMHTS, Police, Criminal Justice and primary care.

Simple entry criteria: 1. Substance Dependence 2. Positive pregnancy test with referral taken from any service. Patients receive comprehensive initial assessment covering addictions, mental health, social circumstances, obstetric history and physical health evaluation including foetal US scanning. Led by a team of psychiatrist, midwife, obstetrician and substance worker.

Evaluation identifies risks from mental, physical health, safeguarding, support needs and formulates an initial engagement and management plan. Referral into all necessary organisations. A staggered follow up plan per every trimester agreed.

Commencement or planned reduction of Opiate Substitution Therapy (OST), medication rationalisation, nutritional advice, enhanced antenatal monitoring. The regular follow-up via fortnightly midwife, drugs worker review. Monthly medial review in the clinic.

The support from perinatal psychiatry teams, CMHTS, Social services, Criminal Justice safeguarding teams is roped in when needed. Child protection, safeguarding issues are addressed. Clear multi-directional communication is maintained at all times. A safe delivery plan along good neonatal management ensured with appropriate outcomes for mother & baby are achieved.

**Results:**

Since 2019, this initiated 16 patients with various complexities. 12 women left hospital with their baby in their care. 1 left the area during the pregnancy. 2 babies were removed into care. 1 had a miscarriage, 1 had a false positive test. All women received contraceptive advice, one got tubectomy and many on long-term contraception. No significant mental health relapses or admissions. All managed to stabilize or reduce their opiates issues.

**Conclusion:**

This One Stop Clinic has effectively addressed the complex needs of perinatal addiction patients. Centralised provision of care, duplication avoided, clear communication was a welcome relief for patients. Clinic has won a quality award.

